# Analysis of Nicotine Metabolites in Hair and Nails Using QuEChERS Method Followed by Liquid Chromatography–Tandem Mass Spectrometry

**DOI:** 10.3390/molecules25081763

**Published:** 2020-04-11

**Authors:** Junhee Kim, Hyun-Deok Cho, Joon Hyuk Suh, Ji-Youn Lee, Eunyoung Lee, Chang Hwa Jin, Yu Wang, Sangwon Cha, Hosub Im, Sang Beom Han

**Affiliations:** 1 Department of Pharmaceutical Analysis, College of Pharmacy, Chung-Ang University, 84 Heukseok-ro, Dongjak-gu, Seoul 06974, Korea; juny456456@naver.com (J.K.); green007n@daum.net (H.-D.C.); velvetrose0805@naver.com (J.-Y.L.); eylee@hanmi.co.kr (E.L.); jeench28@samjinpharm.co.kr (C.H.J.); 2 Department of Food Science and Human Nutrition, Citrus Research and Education Center, University of Florida, 700 Experiment Station Rd, Lake Alfred, FL 33850, USA; joonhyuksuh@ufl.edu (J.H.S.); yu.wang@ufl.edu (Y.W.); 3 Department of Chemistry, Dongguk University, 30 Pildong-ro 1-gil, Jung-gu, Seoul 04620, Korea; chasw@dongguk.edu; 4 Institute for Life & Environmental Technology, Smartive Corporation, Dobong-ro 110 na-gil, Dobong-gu, Seoul 01454, Korea; hosublim@gmail.com

**Keywords:** nicotine metabolites, QuEChERS, bioanalysis, hair, nail, LC–MS/MS

## Abstract

Many studies have analyzed nicotine metabolites in blood and urine to determine the toxicity caused by smoking, and assess exposure to cigarettes. Recently, hair and nails have been used as alternative samples for the evaluation of smoking, as not only do they reflect long-term exposure but they are also stable and easy to collect. Liquid-liquid or solid-phase extraction has mainly been used to detect nicotine metabolites in biological samples; however, these have disadvantages, such as the use of toxic organic solvents and complex pretreatments. In this study, a modified QuEChERS method was proposed for the first time to prepare samples for the detection of nicotine metabolite cotinine (COT) and *trans*-3′-hydroxycotinine (3-HCOT) in hair and nails. High-performance liquid chromatography–tandem mass spectrometry (LC–MS/MS) was used to analyze traces of nicotine metabolites. The established method was validated for selectivity, linearity, lower limit of quantitation, accuracy, precision and recovery. In comparison with conventional liquid-liquid extraction (LLE), the proposed method was more robust, and resulted in higher recoveries with favorable analytical sensitivity. Using this method, clinical samples from 26 Korean infants were successfully analyzed. This method is expected to be applicable in the routine analysis of nicotine metabolites for environmental and biological exposure monitoring.

## 1. Introduction

Direct or indirect smoking is closely related to various diseases, such as cancer and respiratory and cardiovascular diseases. Nicotine is a natural alkaloid contained in the tobacco plant, and a major substance linked to cigarette addiction. Nicotine absorbed into the human body through cigarette smoking can be converted into multiple metabolites [1]. Approximately 70–80% of the absorbed nicotine is transformed to cotinine (COT) by the CYP450 system (mainly CYP2A6). This primary metabolite is further converted into *trans*-3′-hydroxycotinine (3-HCOT) [2], and both COT and 3-HCOT are major metabolites of nicotine. The half-life of nicotine is relatively short, at approximately 3–4 h. In contrast, COT and 3-HCOT have relatively long half-lives of approximately 7–40 h and 4–8 h, respectively, making them suitable alternative indicators of cigarette smoking.

Nicotine and its metabolites have been analyzed using various biological specimens, including urine, plasma and saliva [3]. Keratinic matrices, such as hair and nails, can provide several advantages over the aforementioned biological fluids. They can not only reflect long-term exposure because of their sub-centimeter monthly growth rates, but are also relatively easy to collect and store in comparison with biological fluids. Based on these merits, in forensic studies, clinical applications using hair and nail samples have been actively studied, and several papers on nicotine and its metabolites have been published [4,5,6,7,8,9,10,11,12,13,14,15].

Current analyses of nicotine metabolites in hair and nails are performed using liquid chromatography–tandem mass spectrometry (LC–MS/MS) [5,6,7,9] and gas chromatography–mass spectrometry (GC–MS) [4,16]. In many cases, liquid-liquid extraction (LLE) is employed for sample preparation [4,5,7,9]. Although LLE offers specificity for individual compounds, it may require complicated procedures, as well as highly toxic organic solvents. For example, a method for the determination of four nicotine metabolites employed multiple LLE steps: LLE including primary extraction, and back-extraction followed by re-extraction [9]. Each step used dichloromethane as the extraction solvent. Dichloromethane is a representative chlorinated solvent classified as a possible carcinogen (group 2B) by the International Agency for Research on Cancer (IARC) [17].

The QuEChERS (quick, easy, cheap, effective, rugged and safe) sample preparation method has been widely applied to multi-component analysis, such as pesticides, animal drugs and fungal poisons in various matrices, based on its advantages of low price, simplicity and high selectivity, as well as its minimal usage of harmful chemicals [18,19]. To the best of our knowledge, there is no case of QuEChERS being applied in analysis with nails as a target matrix. It is also difficult to find examples of the use of QuEChERS to prepare hair samples. Although a hair-based QuEChERS study was reported, the target analytes were limited to pesticides and psychoactive drugs [20,21]. However, considering its ease of operation and safety, it is expected to be advantageous in its application to keratinic matrices.

In this study, a new approach using QuEChERS to detect nicotine metabolites in hair and nails was proposed. We aimed to simplify the sample preparation procedure and avoid the use of toxic solvents such as dichloromethane. The established method was validated according to the relevant guideline [22], and its potential for clinical application was verified by monitoring 26 clinical hair and nail samples.

## 2. Results and Discussion

### 2.1. Optimization of Sample Preparation

**Figure 1:**
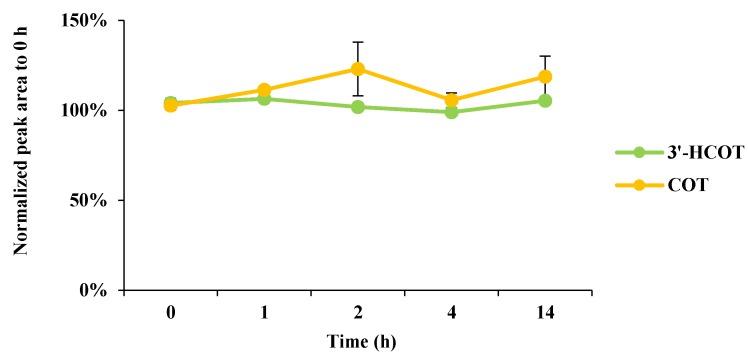
Effect of digestion time in 1 M NaOH on stabilities of COT and 3-HCOT (*n* = 3). Peak areas were normalized with respect to the corresponding peak areas obtained at 0 h.

For hair and nail samples, which may be easily contaminated by external sources, a washing step was necessary for accurate analyte measurement. The efficiency of the washing agents was tested using a 0.1% sodium dodecyl sulfate (SDS) aqueous solution, methanol, or a combination of the two. Two factors were considered to select the best wash solvent: maximizing the washing efficiency of the contaminated target analytes from external sources and minimizing the loss of residual authentic analytes in the target matrix. The washing efficiency of externally derived components and the loss of residual components were assessed by varying the exposure time to washing solvents: 1 min for the former and 30 min for the latter. In terms of washing external cotinine (COT) and *trans*-3′-hydroxycotinine (3-HCOT), the 0.1% SDS solution exhibited a capacity similar to that of methanol. On the other hand, in the long-term exposure, there was a difference between 0.1% SDS solution and methanol in terms of the loss of residual COT in a hair sample: 0.1% SDS solution lost little or no COT, but methanol lost approximately 30% (data not shown). Hence, 0.1% SDS solution was chosen as the washing solvent for the monitoring samples, and methanol was used as the washing solvent for the preparation of blank samples. Next, for sample digestion, strongly acidic (1 M hydrochloric acid, HCl) and basic (1 M sodium hydroxide, NaOH) solutions were evaluated with or without heating (90 °C). The 1 M NaOH solution digested samples better than the 1 M HCl solution. The recoveries of COT and 3-HCOT were significantly low under thermal treatment, due to stability issues (data not shown). Thus, 1 M NaOH was selected for the digestion, and the procedure was performed at room temperature (20–25 °C). The stability of the analytes over different digestion times (0–14 h) was also examined; both analytes are stable under all conditions (Figure 1).

**Figure 2:**
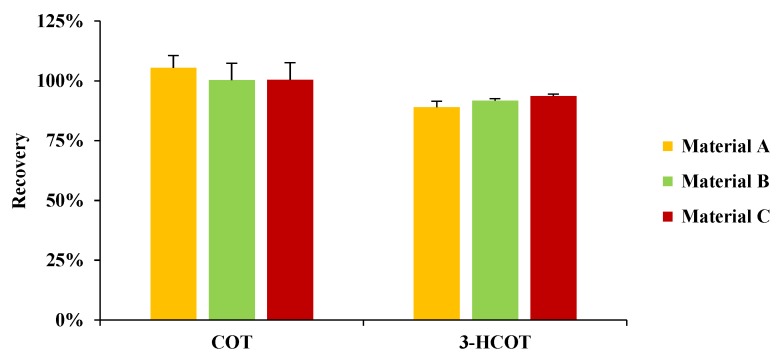
Effect of different materials for QuEChERS on the extraction recovery of COT and 3-HCOT (*n* = 3): material A (magnesium sulfate (800 mg), sodium acetate (200 mg) and primary secondary amine (PSA, 150 mg)); material B (magnesium sulfate (800 mg), sodium chloride (200 mg) and C18 powder (150 mg)); and material C (magnesium sulfate (800 mg), sodium chloride (200 mg) and PSA (150 mg)).

Finally, the QuEChERS technique (including dispersive solid-phase extraction, SPE) was tested for the extraction of COT and 3-HCOT using different materials: material A (magnesium sulfate (800 mg), sodium acetate (200 mg) and primary secondary amine (PSA, 150 mg)) [23]; material B (magnesium sulfate (800 mg), sodium chloride (200 mg) and C18 powder (150 mg)); and material C (magnesium sulfate (800 mg), sodium chloride (200 mg) and PSA (150 mg)). As illustrated in Figure 2, all materials exhibit good extraction efficiencies. However, traces of COT are detected in the sodium acetate in material A and the C18 powder in material B. Thus, material C was chosen for the QuEChERS sample preparation.

### 2.2. Method Validation

**Figure 3:**
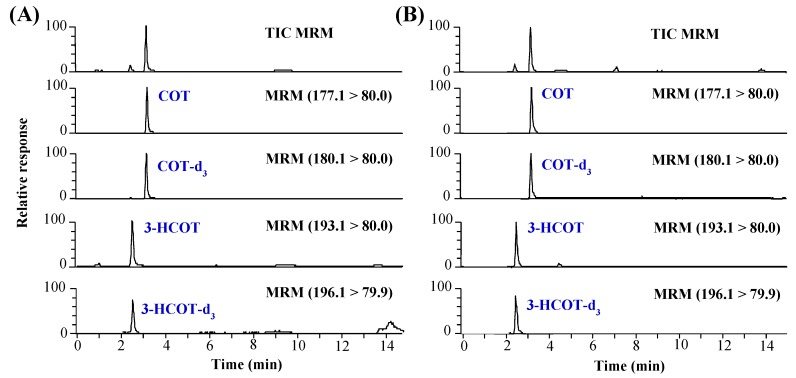
LC–MS/MS chromatograms of target analytes and internal standards spiked in: (**A**) blank hair; and (**B**) blank nail samples.

The developed method was validated in terms of selectivity, linearity, lower limit of quantitation (LLOQ), accuracy, precision and recovery [22]. As depicted in Figure 3, no interference is observed at the retention times of COT (3.3 min) and 3-HCOT (2.6 min) when each blank sample is analyzed. The calibration standards were prepared by spiking target analytes into blank (target analyte-free) hair and nails at six points in the range of 10–9,000 pg/mg (10, 30, 100, 300, 1500 and 9000 pg/mg). The regression coefficients (*r^2^*) of the calibration curves are higher than 0.999 over the range in both hair and nail samples. The LLOQs, defined as the concentration producing a signal-to-noise (*S/N*) ratio of 5, are 10 pg/mg for both COT and 3-HCOT in the hair and nail samples. The accuracy and precision were determined at four different concentrations (10, 30, 300 and 9000 pg/mg) within the calibration range (*n* = 5 for intra-day and *n* = 3 for inter-day). The accuracies are 93.3–105.0%, and the precisions (represented by the relative standard deviation, RSD) range from 0.3% to 12.3%, where all values are within the acceptable range. The recovery was investigated by the standard addition method at three different levels (30, 300 and 9000 pg/mg) in triplicate. The mean recoveries are 63.6–86.3%. The method validation results are summarized in Table 1.

### 2.3. Method Application

The developed method was applied to 26 clinical samples obtained from infants. Using these samples, we intended to verify that hair and nails can be used to assess second-hand smoke exposure, and determine whether the established method can detect nicotine metabolites in real samples. As presented in Table 2, out of the 26 samples, the number of hair and nail samples in which COT is detected are ten and eight, respectively, and 3-HCOT is detected in just two samples of both hair and nails. Based on these results, we estimate that COT is more suitable than 3-HCOT as a biomarker for indirect exposure to nicotine in the keratinic matrices. The median COT concentrations for hair and nails are 37.6 pg/mg (10.2–1157.2 pg/mg) and 13.7 pg/mg (10.0–21.4 pg/mg), respectively. This presumably results from the different deposition mechanisms of the nicotine metabolites in hair and nails, which accumulate in different amounts. A limitation of this study is that no information was provided on whether the subjects’ parents smoked, which makes it difficult to interpret the extent of metabolic accumulation caused by smoking. Further research will be needed to determine the relationship between the parents’ smoking habits and the accumulation of nicotine metabolites in their children’s hair and nails. However, the developed method successfully analyzes the target components in the hair and nail samples, which recommends its clinical use.

### 2.4. Comparison with Other Methods

The proposed method was compared with a previous method using LLE [4,5,7,9]. The same amount of sample (10 mg) was used for each preparation method. As presented in Table 3, our method resulted in higher recoveries, particularly for 3-HCOT, with lower RSD values than the LLE method. The highly toxic solvent dichloromethane was replaced with acetonitrile. Indirect comparisons with other studies using different extraction techniques [4,5,7,9] reported in Table 4 also supported the enhanced ability of our method.

## 3. Materials and Methods 

### 3.1. Reagents and Materials

Cotinine (COT), *trans*-3′-hydroxycotinine (3-HCOT), cotinine-*d*_3_ (COT-*d*_3_), *trans*-3′-hydroxycotinine-*d*_3_ (3-HCOT-*d*_3_), sodium dodecyl sulfate (SDS) and DSC-18 solid-phase extraction (SPE) powder were purchased from Sigma-Aldrich (St. Louis, MO, USA). Acetonitrile, methanol and water were of liquid chromatography–mass spectrometry (LC–MS) grade and purchased from Fisher Scientific (Fair Lawn, NJ, USA). Sodium hydroxide, magnesium sulfate, sodium chloride and sodium acetate were acquired from Daejung Chemical & Metals Co. (Siheung, Republic of Korea). Primary secondary amine (PSA) bulk sorbent was purchased from Agilent Technologies (Santa Clara, CA, USA). All other reagents were of analytical grade.

### 3.2. Preparation of Standard Solutions and Human Hair and Nail Samples

Stock solutions of COT (1,000 µg/mL), 3-HCOT (100 µg/mL) and internal standards (IS; COT-*d*_3_ and 3-HCOT-*d*_3_; and 100 µg/mL) were prepared using methanol and stored at -20 °C. Working solutions were prepared daily by diluting and mixing each stock solution with methanol prior to use. Calibration standards were prepared at proper concentrations by diluting the working solution with blank hair or nail samples. Clinical samples were collected from 26 Korean infants, and kept at room temperature. Blank samples were prepared by washing the samples with methanol (agitation for 30 min, three times) after homogenization. This study was approved by the Institutional Review Board of Chung-Ang University (IRB No. 1041078-201709-BR-179-01).

### 3.3. Sample Preparation

The segments of hair and nail samples were washed with a 0.1% SDS aqueous solution (25 mL) by agitation for 30 min. After drying, the samples were finely cut using a pair of scissors. A portion of the samples (10 mg) was digested in a 1 M NaOH aqueous solution (1 mL) overnight at room temperature. Acetonitrile (5 mL) and the IS solution (10 μL) were added to the resulting sample, and the mixture was vortexed for 15 min. The sample was further agitated for 20 min with the QuEChERS (quick, easy, cheap, effective, rugged and safe) salts (800 mg magnesium sulfate and 200 mg sodium chloride), and then centrifuged at 2800 g for 10 min. The organic phase was transferred into a second tube and PSA (150 mg) was added. The mixture was vortexed for 15 min and centrifuged at 2800 g for 10 min. The supernatant was dried under a nitrogen stream at room temperature, and the dried residue was reconstituted with a water/methanol/acetonitrile mixture (0.3 mL, 70/15/15, v/v/v). The solution was filtered using a polytetrafluoroethylene syringe filter (0.2 μm), and a portion of the filtrate (5 μL) was injected into the LC–MS/MS.

### 3.4. LC–MS/MS Analysis

The LC–MS/MS assay was performed using an Agilent 1290 LC system coupled to an Agilent 6490 triple quadrupole MS detector. The LC separation was performed using a Phenomenex (Torrance, CA, USA) Kinetex C18 column (2.1 mm × 100 mm, 2.6 μm particle size) at 30 °C. The mobile phase consisted of (A) water and (B) methanol/acetonitrile (50/50, v/v), with a gradient elution as follows: 0–8 min, 10–90% B; 8–12 min, holding at 90% B; 12–12.1 min, 90–10% B; and 12.1–15 min holding at 10% B. The flow rate was 0.2 mL/min and the injection volume was 5 µL. The mass spectrometer was operated in the multiple reaction monitoring (MRM) mode with positive electrospray ionization (ESI^+^). Nitrogen gas was used as the nebulization, desolvation and collision gas. The optimized ESI conditions were as follows: capillary voltage, 3000 V; drying gas flow, 16 L/min; drying gas temperature, 290 °C; nebulizer, 25 psi; sheath gas temperature, 400 °C; sheath gas flow, 12 L/min; nozzle voltage, 500 V; and ion funnel voltage pressure, 60–150 V. Optimum MRM transitions of the analytes are presented in Table 5. 

## 4. Conclusions

A QuEChERS (quick, easy, cheap, effective, rugged and safe) method combined with liquid chromatography–tandem mass spectrometry (LC–MS/MS) analysis for the detection of the nicotine metabolites cotinine (COT) and *trans*-3′-hydroxycotinine (3-HCOT) in hair and nail samples was proposed for the first time. The method was optimized, validated and applied to real samples obtained from infants. The proposed method demonstrated favorable sensitivity, and was proven to be simple and more efficient in comparison with previous methods based on liquid-liquid extraction (LLE). Moreover, the avoidance of chlorinated solvents increases this method’s appeal. We believe that this method can be effectively applied to the routine analysis of nicotine metabolites in biological samples for various purposes.

## Figures and Tables

**Table 1:** Validation summary for the proposed method.

Validation Parameters	Hair		Nail	
	COT	3-HCOT	COT	3-HCOT
Linearity				
Range (pg/mg)	10–9000	10–9000	10–9000	10–9000
*r^2^*	0.9999	0.9999	0.9999	0.9999
LLOQ (pg/mg)	10	10	10	10
Precision (RSD %, four different levels)				
Intra-day (*n* = 5)	2.1–9.6	0.6–8.2	1.1–3.1	1.3–4.3
Inter-day (*n* = 3)	1.7–4.9	1.3–4.1	0.3–12.3	1.0–9.7
Accuracy (%, four different levels)				
Intra-day (*n* = 5)	93.3–101.2	95.6–102.9	97.0–102.9	97.7–104.2
Inter-day (*n* = 3)	94.0–105.0	95.2–103.9	96.7–100.5	97.0–102.7
Recovery (%, *n* = 3)				
Low level	85.1	83.8	78.2	65.3
Medium level	87.2	71.3	70.4	57.1
High level	86.5	72.8	78.2	68.5

**Table 2:** Summary of monitoring results of nicotine metabolites in 26 infant samples.

	Hair		Nails	
	COT	3-HCOT	COT	3-HCOT
**Number of detected samples**	10	2	8	2
**Median concentration (pg/mg)**	37.6	182.9	13.7	10.6
**Interquartile range (pg/mg)**	1147.0 (10.2–1157.2)	320.8 (22.4–343.3)	11.4 (10.0–21.4)	0.8 (10.2–10.9)

**Table 3:** Comparison of the proposed method with the previous LLE method [11].

Method	Organic Solvent (Volume)	Analyte	*S/N* ratio	Recovery (*n* = 3)	
				Mean	RSD
QuEChERS	Acetonitrile (5 mL)	COT	7329.6	83%	2%
		3-HCOT	344.5	61%	1%
LLE	Dichloromethane (2 mL)	COT	22,227.0	65%	11%
		3-HCOT	68.4	11%	16%

**Table 4:** Comparison of the proposed method with other methods for the detection of nicotine metabolites.

Sample (Amount)	Analyte	Sample Preparation	Extraction Step ^a^	LOD ^b^ (pg/mg)	Ref.
Hair (10 mg)	COT	LLE	Extraction with DCM (0.5 mL)	6.6	[7]
Hair (1 mg)	COT	LLE	Extraction with diethyl ether (2 mL)	70	[24]
Hair (20 mg)	COT	LLE	Three step extraction: - Extraction with DCM (5 mL) and DCM/iPrOH (5 mL, 75/25, v/v) - Back-extraction with HCl (1 mL, 0.5 M) - Re-extraction with DCM (4 mL) and DCM/iPrOH (4 mL, 75/25, v/v)	2.5	[9]
Toenail (20–30 mg)	COT	LLE	Extraction with DCM (1 mL)	12	[25]
Toenail (20–30 mg)	COT	LLE	Repeated extraction (thrice) with DCM (0.5 mL)	35	[4]
Hair and nail (10 mg)	COT, 3-HCOT	QuEChERS	One-step extraction with acetonitrile (5 mL)	10 ^c^	This work

^a^ Dichloromethane (DCM) and isopropanol (iPrOH). ^b^ Limit of detection (LOD). ^c^ Represented as LLOQ.

**Table 5:** MRM transitions and collision energies for target analytes and internal standards.

Analyte	*t_R_*^a^ (min)	Precursor Ion (*m/z*)	Product Ions (*m/z*)		
			Quantitative Ion (CE ^b^)	Qualitative Ions (CE)	
3-HCOT	2.6	193.1	80.0 (30)	134.1 (20)	106.0 (29)
3-HCOT-*d*_3_ (IS)	2.6	196.1	79.9 (32)	134.0 (19)	106.0 (31)
COT	3.3	177.1	80.0 (29)	98.0 (21)	70.1 (34)
COT-*d*_3_ (IS)	3.3	180.1	80.0 (28)	101.0 (23)	73.1 (39)

^a^ Retention time (*t_R_*). ^b^ Collision energy (CE).
